# How is the balance between protein synthesis and degradation achieved?

**DOI:** 10.1186/1742-4682-7-25

**Published:** 2010-06-23

**Authors:** Stephen Rothman

**Affiliations:** 1 University of California, San Francisco, San Francisco, CA 94143, USA

## Abstract

Unlike most substances that cells manufacture, proteins are not produced and broken down by a common series of chemical reactions, but by completely different (independent and disconnected) mechanisms that possess no intrinsic means of making the rates of the two processes equal and attaining steady state concentrations. Balance between them is achieved extrinsically and is often imagined today to be the result of the actions of chemical feedback agents. But however instantiated, chemical feedback or any similar mechanism can only rectify induced imbalances in a system *previously balanced by other means*. Those "other means" necessarily involve reversible mass action or equilibrium-based interactions between native and altered forms of protein molecules somewhere in time and space between their synthesis and degradation.

## Introduction

While developing successful all-encompassing or general models to account for life's properties is the hope of much scientific research in biology, life's varied and complex nature at times seems to preclude easy generalization. Protein metabolism, the events that make and degrade proteins as well as the mechanisms that regulate the rates of these processes, is a case in point. Not only is each protein, for instance the many thousands of different kinds manufactured by eukaryotic cells, structurally and functionally unique, so is the path, variety, variability, and duration of their life history. After synthesis, some undergo major physical and chemical changes for reasons as varied as the changes themselves, while others seem to remain essentially unchanged. In the process of change they may be added to or reduced in size, or they may be modified time and again as they perform a continuing function. In addition, some are destroyed almost as rapidly as they are made, while others last a lifetime, or as in growing bacterial cultures are only broken down when cell division ceases or as with the enucleate red blood cell when the cells that contain them are destroyed or as with the apoprotein of the retina in the order in which they are made. In yet other cases, for instance as part of an immune response or during development, they are only expressed for brief periods of time under very particular circumstances. The complexities of the life history of proteins are enormous, as or more complex than the structure of these most complicated of molecules, and in some respects matches, perhaps unsurprisingly the complexity of life itself.

Given such facts, despite the enormous experimental knowledge base about the production and destruction of proteins, it is not surprising that the important question about protein metabolism posed in this paper's title, "How is the balance between protein synthesis and degradation achieved?" has not only not been answered, to the best of my knowledge it has never been explicitly asked. This even though in the fullness of time balance between the rates of manufacture and destruction, between what is made and what is broken down occurs and is quantitative whatever the protein, however and wherever degradation takes place, and even though most proteins in eukaryotes, in both the cellular and extracellular compartments of metazoans, as well as in non-growing bacterial cultures, are present at stable and reproducible concentrations for a given physiological steady state, signifying balance between their rates of synthesis and breakdown. Furthermore, when changes in concentration occur, due either to altered physiological circumstances or the presence of disease, a new steady state concentration is usually sought and found.

But the absence of discussion should not be taken to mean an absence of opinion. Thomas Kuhn in describing the nature of the scientific paradigm argued that there are really no open questions, or at least no open questions of significance in scientific disciplines. Whether supported by evidence and reason or merely expressions of bias, the paradigm leaves no question unanswered, even if only implicitly. In this regard, things are particularly difficult for protein metabolism. Because proteins are central to virtually every area of biology, from molecular biology, to biophysics, to structural biology, to microbiology, to biochemistry, to cell biology, to immunology, to pathology, to physiology and systems biology, there are often different, non-commuting disciplinary perspectives. In this regard, in what follows we will consider lysosomal degradation, feedback regulation, and the equilibration of native and altered proteins as potential answers to the question posed in the article's title.

In any event, taken together such circumstances are not only ripe for strong differences of opinion, but make attempts to generalize about how balance is achieved daunting. And yet, science cannot simply demur and decide that the question not only can't be answered, it shouldn't be asked, or that asking it is a pointless or fruitless exercise. It is duty bound to seek broad explanatory rules however seemingly complex and varied the phenomena. The analysis that follows is based on fidelity to this belief, with appreciation for the difficulty of the task at hand and awe at life's still unexposed mysteries.

## Background

With some exceptions such as growing bacterial cultures, even a small persistent imbalance between the rates of synthesis and degradation of proteins is inimical to cellular and organismal life^1^. Over the past half-century we have learned a great deal about how proteins are manufactured and degraded, the rates at which they turn over, and how these processes are regulated. However, little attention has been paid to how balance, or parity, between the two is achieved.

For most substances that cells manufacture their rate of formation, or anabolism, and the rate of their breakdown or transformation, or catabolism, are balanced by mass action, expressed in common or related chemical reactions and intermediate states (e.g., A + B <--> C <--> D + E). Things are entirely different for proteins. Most importantly, the mechanisms responsible for their manufacture and breakdown are not part of a common chemical process, but are completely independent of each other both chemically and physically. In addition, and also unlike other molecules, the rates at which these processes occur is not determined by the rate at which the chemical bonds that form the substances are made or broken, but by external factors, for example, the amount of mRNA for synthesis or the rate of ubiquitination for degradation [1,2].

Finally, both synthesis and degradation are irreversible processes in that they are unresponsive to mass action effects of their end products (proteins and amino acids respectively) on their rates. For example, if we take a protein and break it down to its substituent amino acids, not even a small amount will reassemble spontaneously. Protein synthesis is the most expensive biosynthetic process known to us, and reconstruction in the absence of a great deal of free energy is extremely unlikely, but even if the energy were available, without a means of generating the appropriate sequence of amino acid subunits, as is done by mRNA during synthesis, the authentic peptide chain simply cannot be reconstituted.

Nor is a mass action effect of a protein on its own rate of synthesis any more likely. Once manufactured, the new protein is released from the synthetic machinery of the ribosome into the cytosol or other cellular compartment. As such, it cannot affect upstream events on the ribosome by mass action. Indeed, there are no upstream events to affect. Ribosomes are assembly lines for the construction of *single *peptide chains [3,4]. As the nascent chain moves through the ribosomal machinery, no other chains are being produced behind it on the same ribosome. The process is discontinuous, and after a new protein is discharged, the ribosome becomes inactive. Its two major subunits dissociate until a new mRNA molecule comes along to start the process over again, in all likelihood for a different protein.

## Lysosomal degradation

According to one line of current thinking, there are two general mechanisms for the degradation of proteins in eukaryotic cells, one for cytosolic and nuclear proteins, and another for proteins that are contained in or are part of large intracellular structures (excepting the nucleus), such as various membrane-enclosed vesicles and organelles. For cytosolic and nuclear proteins, breakdown occurs within proteasomes, small freestanding pore-like aggregates of degradative enzymes and regulatory proteins found in the cytosol and nucleoplasm [5-10]. It is thought that dysfunctional structural changes occur to protein molecules over time due to random environmental causes, or as a result of being defective initially, and that as a consequence certain exposed regions on the altered molecules serve as the predicate for their degradation. For at least some, a small protein, ubiquitin, affixes to particular imperfections and marks them for destruction [11-16]. In the other degradative system, entire anatomical structures enter small membrane-enclosed sacs known as *lysosomes *as the result of membrane fusion [17-19]. Subsequent to fusion, lysosomal enzymes disassemble and degrade the structure and its contents, including its proteins.

While the proteasome system appears capable of achieving metabolic balance (see below), the lysosomal system does not. Though lysosomes may disassemble and degrade foreign bodies and their contained chemicals [20], or be responsible for autophagic responses to cellular pathology and aging [21], they seem ill suited to balance the ongoing manufacture and degradation of endogenous proteins. There are two reasons.

First, by necessity the rate-limiting step in lysosomal degradation is membrane fusion. Otherwise, the fused objects would continuously accumulate in the cell in anticipation of processing. This does not normally occur, and cellular life could not be sustained if it did. As a consequence of fusion being the rate-limiting step, all of the proteins in a given structure would have to be degraded and, to achieve balance, synthesized at a single common rate, the rate of fusion. This is not the case. Different proteins are made and degraded, turned over, at distinctive and often quite disparate rates even when present in the same structure.

The second reason is even more compelling. To achieve balance cells would actually have to *know *the rate of fusion, as well as the protein contents of the fused objects, the mathematical product of the two, and have the means to transmit this information to the synthetic machinery. As we understand biological cells, such tasks lie beyond their capabilities. They have no more knowledge of what they are doing than clouds or rivers. In any event, in what follows I only consider proteins that are broken down in proteasomes, even though the general requirements for producing metabolic balance apply whatever mechanism is employed^2^. In addition, I only take into account proteins that are present at stable values for particular physiological steady states.

## The effect of feedback

**Figure 1:**
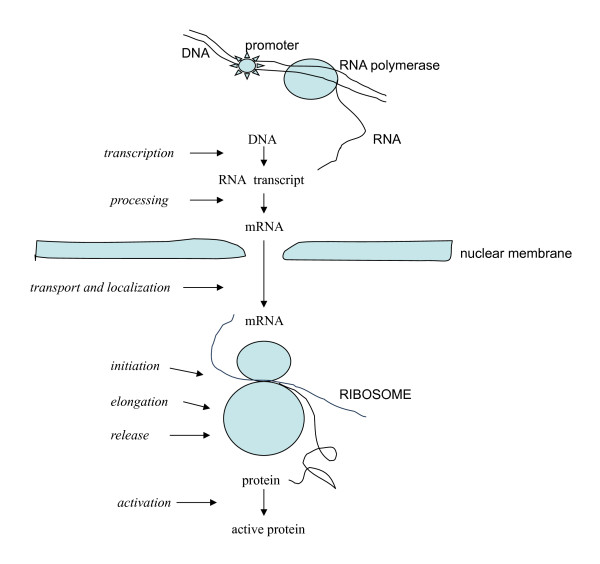
**The feedback regulation of protein synthesis**. Shown are events of protein synthesis that are affected either directly or indirectly by feedback agents (italics)(see *The effect of feedback*).

**Figure 2:**
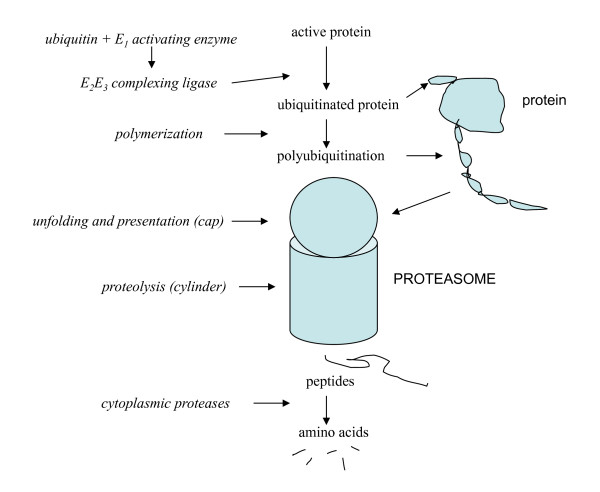
**The feedback regulation of protein degradation**. Shown are events of proteasomal degradation (for an ubiquitination system) that are affected either directly or indirectly by feedback agents (italics)(see *The effect of feedback*).

If the mechanisms that determine the rate at which a protein is made and those that determine the rate at which it is broken down possess no intrinsic means of making the two equal, then balance between them requires a mechanism that is *extrinsic *to these processes. As said, this is usually, though not uniformly, imagined today in terms of chemical feedback. Chemical agents or signals, acting separately or together, synergistically or antagonistically, on one process or both, feedback on various steps in synthetic and degradative pathways adjusting their rates to achieve metabolic balance (figures 1 and 2)[1,2].

For synthesis, the feedback agents alter the *production of mRNA *from its DNA template (transcription)[1,2,22-25], as well as its *availability and effectiveness *subsequently (post-transcriptionally)(figure 1)[26-30]. For degradation they in the main act on the events that immediately *precede *breakdown, that is, on choosing or preparing molecules for degradation (figure 2)[1,2,31-38].

And yet unlike chemical reactions where mass action produces balance between production and breakdown automatically as the reaction seeks a steady- or equilibrium state, for a negative feedback agent or other extrinsic mechanism to achieve balance requires something quite different and as it happens quite unlikely. That is, the mathematical product of its concentration and the avidity with which it binds to relevant sites must combine to alter the rate of manufacture or breakdown of the substance by just the right amount so that by fortunate circumstance it equals the rate of the countervailing process. For example, if the feedback agent were the end product of an enzyme-catalyzed chemical reaction, its concentration and the avidity with which it binds to the catalyst would have to combine to produce a change in the catalyst's effectiveness that by chance would alter the reaction's rate to the same degree that it would have been altered if the end product had acted by mass action. For the transcriptional regulation of protein synthesis, this would require that a particular concentration of a feedback agent bind to a regulatory protein with an avidity that produces a concentration of the resultant complex that binds to DNA with just the right affinity so that in repeated acts of association and dissociation, transcription is turned on and off at a frequency that produces an amount of mRNA that yields a rate of protein synthesis equal to that of degradation [1]. Such a concatenation of events seems implausible.

Given the unlikelihood of these and analogous circumstances, chemical feedback, whatever its incarnation, can only rectify induced imbalances *in a system already balanced by other means*. It cannot establish that balance in the first place. For example, if an increase in the concentration of a protein occurs due to an elevation in its rate of synthesis, feedback can produce a proportional increase in the rate of degradation, forcing a return to a prior concentration and state of balance. If however the two rates were unequal initially, increasing them in proportion to each other would not make them equal. In this case, if a = b, then 2a = 2b, but if a > b, then 2a would remain greater than 2b (2a > 2b)^3^.

## Protein turnover

To establish, as opposed to re-establish balance between synthesis and degradation, a different sort of mechanism is needed. An important clue to that mechanism was discovered many years ago in studies on *protein turnover *-- the renewal and replacement of proteins [39-45]. In this large body of work, a protein's rate of turnover was commonly estimated by producing a dislocation from a prior steady state concentration by increasing or decreasing its rate of synthesis or degradation artificially and then passively observing as a new steady state was approached, or by following the disappearance of radio-labeled proteins from previously labeled cells and tissues, or by measuring the incorporation of radioactive amino acids into cellular protein. The mathematical functions that described these phenomena exposed the kinetic nature of the mechanisms that produce balance, though they did not disclose their details.

As expected, given its irreversible character, synthesis was a zero-order or linear process. But degradation, also understood to be irreversible, was not. Its kinetics was first-order or exponential, and suggested a reversible or equilibrium-dependent event. Given what I said about the irreversibility of degradative processes, this is deeply contradictory. We would expect degradation to be a zero-order process, just like synthesis. And yet this said, what was observed was entirely predictable. If both synthesis and degradation were zero-order or linear processes, life could not be sustained. Since linear functions do not converge, any difference in the rates of synthesis and degradation would continue *ad infinitum*; there could be no equilibration, no steady state. Of course, degradation occurring more rapidly than synthesis is a *non sequitur *since the cell would be void of the protein forever, but assuming that the rate of synthesis is greater, the concentration of the substance would rise monotonically and ceaselessly over time.

The contradiction that this irreversible chemical process is of the first order can only be resolved if the first order kinetics are *not *attributable to degradation itself, but to a foregoing process that sets its rate. This foregoing process must be reversible, in other words equilibrium-based mass action. That this is so is indicated variously by the chemical or reaction-based nature of turnover, the first-order isotope kinetics seen at the steady state (when production and degradation are equal), and the tracer kinetics of isotope incorporation studies^4^. As a consequence, degradation need not impossibly be both reversible (first-order) and irreversible (zero-order) at one and the same time. It is an irreversible process whose rate is regulated by a preceding reversible event.

## Equilibration

This reflects a primary causal discernment. As a general matter, chemical events are balanced by mass action among their constituents, and despite the unique circumstances of protein synthesis and degradation, physical law does not allow an exception to this rule; it provides no other means of achieving balance. The question then is not whether this occurs, but how and where? If balance is not achieved within the synthetic and degradative reaction sequences in their own right, and it is not, then it must take place external to them. That is, the mass action event must occur somewhere in time and space between the manufacture of proteins on ribosomes and their breakdown in proteasomes, in other words in the solvent phases of the cell that contain both of these structures^5^.

Among which molecules would this mass action effect occur? Based on the understanding that each protein turns over at a unique rate, equilibration must be between the "native" protein -- physiologically capable or mature forms of the molecule and modified or altered forms of the same protein that are *predisposed to degradation *and that set its rate. The equilibrium constant between the two forms reflects their ratio in the cell at the steady state. In this way, the rates of the irreversible and independent mechanisms of synthesis and degradation are joined and balanced both in the first instance and subsequently.

## Evidence

While the existence of such an equilibration, however it is executed, is as true as the assumption that the synthesis and degradation of proteins are equal at the steady state, as with any theoretical conclusion experimental validation is important. As such, we should ask whether there is evidence for the predictions of the inference and where there is none, is it susceptible to experimental verification?

Regarding the evidence on hand, three important predictions have not only been validated, but are well established. First, research, most importantly on the ubiquitin system [11-16] as well as on defective (DRiPS) proteins [46-51], has shown that many proteins are altered after their synthesis in ways that predispose them to degradation. Second, turnover studies demonstrate that the rate of degradation is indirectly driven by the concentration of the protein substrate. And finally, also from turnover studies, the first order kinetics of degradation, combined with the irreversible nature of the degradative process, is proof of equilibration prior to degradation.

Together these facts provide substantial validation for the proposal -- protein molecules exist that are predisposed to degradation, the rate of degradation is driven by concentration, and equilibration occurs between different forms of the protein prior to its degradation. All that is missing, and this is not to minimize it, is evidence showing which molecules that are predisposed to degradation are reversibly related to a native form. That is, which particular molecular variants in the chain of events from synthesis to degradation equilibrate? The challenge in obtaining this evidence is not methodological [equilibration can be studied in many ways, in cell free systems, as well as in vivo (e.g., by inhibiting degradation)], but in determining which molecules are involved. And at least initially, finding the relevant molecules may be more a matter of trial and error, than *a priori *determination.

## Equilibrating pools of protein

Beginning with the pioneering work of Wheatley in the 1980s [46-51], we learned that protein synthesis is error-prone. On average 30% (in some cases as high as 90%) of new protein is defective and improperly folded. Even for proteins transferred into the endoplasmic reticulum (ER) as they are synthesized, the defective molecules are transported back into the cytosol to be degraded by the ubiquitin/proteasome system. This transfer is not an oddity, but evidence of a more general fact.

If we exclude lysosomal degradation as a means of achieving balance, absent some yet undiscovered mechanism, all proteins, including those embedded in membranes and contained within intracellular membrane-enclosed structures, must have access to the proteasome system in the cytoplasm or nucleus to be degraded^6^. For this to occur, they must have cytosolic (or nuclear) compartments. But more than that, for turnover to apply to the whole cellular pool, as it ultimately must, this compartment must be in equilibrium with the remainder of its cellular contents wherever they are located. In accordance with this conclusion, Rock, et al found that inhibition of proteasome function produces the almost complete inhibition of protein degradation [52].

## Conclusion

Some of the description given above of the various processes involved in protein metabolism, of synthesis, degradation and their regulation, has of necessity been abbreviated and in many areas lacks details that are no doubt important to specialists. This is for reasons of space -- the details of fact and evidence in the many fields involved is truly enormous -- and clarity -- the belief that extraneous detail would obscure otherwise relatively straightforward concepts. Whatever problems these deficiencies of detail and subtlety introduce, they do not make the presentation less salutary, the issues less cogent or the conclusions less clear. The conclusions, whether about lysosomal degradation, feedback regulation, or most importantly about equilibration, tell us, independent of mechanistic details, what must occur and what cannot occur as a matter of logic and our understanding of physical and chemical kinetics.

The principal conclusion to be drawn from this analysis is that through the agency of mass action and the conservation of mass, the equilibration of complementary forms of the same protein molecule sets and balances its rate of synthesis and degradation. The difference between proteins and most other bioorganic molecules in achieving this balance is that for proteins the mass action effect occurs, in time and space, between their production and breakdown, not as part of it. As explained, physical law requires a means of equilibration, and the separate nature of synthetic and degradative mechanisms by necessity place this event in the solvent phases of the cell that contain ribosomes and proteasomes.

Though turnover studies in the past and the more recent discovery of the ubiquitin pathway provide important evidence for the presence of this equilibration, the current analysis makes it clear that equilibration cannot be part of degradation *per se*, and that the ubiquitin or any foregoing pathway must include an equilibrating element. If attitudes are not too hardened, the assessment presented in this article can serve as a helpful starting point for further exploration of this important, but long ignored subject.

## Competing interests

The author declares that he has no competing interests.

## Appendices

### Appendix 1

A small uncompensated difference, say 1%/day, between the rates of synthesis and degradation of a protein in a cell with a longevity of a year, say a liver cell, would produce enormous and unsustainable changes in its concentration during the cell's lifetime. This said, whether in cells or extracellular fluids, most proteins are found at characteristic steady state or stable values for specified functional states, even in non-dividing bacterial cultures. For example, in a large-scale multivariate study in E. coli, balance between protein production and destruction was found for many proteins under all sorts of conditions [53,54].

### Appendix 2

In this light, inhibition of proteasome function produces the almost complete inhibition of protein degradation [52].

### Appendix 3

For all equilibrium-based physical states and chemical reactions, proteins included, the relationship between the rate constants for synthetic and degradative reactions are proportional or multiplicative (Ks/Kd) [most simply, dP_x_/dt = (Ks/Kd) P_x_(t), where P is the amount of protein x]. In feedback and other similar mechanisms, the constants are related in a subtractive fashion [for kinetics of the same order Ks - Kd or for different orders Ks - *f *(Kd)], and as such balance can only be achieved at a particular invariant concentration (when the difference is zero).

### Appendix 4

These facts eliminate the possibility of an irreversible first-order process analogous to isotopic decay.

### Appendix 5

If a protein's concentration were to directly drive the rate of degradation to match that of synthesis without the intercession of a reversible mass action process, the kinetics of degradation would be zero-order, and as explained this is not the case. Also, concentration is a measure of the *difference *between the rates of formation and breakdown, not their separate and distinctive magnitudes, and as such cannot be used to establish balance between them.

### Appendix 6

Some degradative enzymes are found in the mitochondrion and it is possible that at least some mitochondrial proteins are degraded locally.
